# Identification of Metabolites of Eupatorin in Vivo and in Vitro Based on UHPLC-Q-TOF-MS/MS

**DOI:** 10.3390/molecules24142658

**Published:** 2019-07-23

**Authors:** Luya Li, Yuting Chen, Xue Feng, Jintuo Yin, Shenghao Li, Yupeng Sun, Lantong Zhang

**Affiliations:** 1 School of Pharmacy, Hebei Medical University, Shijiazhuang 050017, China; 2 School of Pharmacy, Hebei University of Chinese Medicine, Shijiazhuang 050000, China

**Keywords:** eupatorin, UHPLC-Q-TOF-MS/MS, metabolism, in vivo *and in vitro*, rat liver microsomes, rat intestinal flora

## Abstract

Eupatorin is the major bioactive component of Java tea (*Orthosiphon stamineus*), exhibiting strong anticancer and anti-inflammatory activities. However, no research on the metabolism of eupatorin has been reported to date. In the present study, ultra-high-performance liquid chromatography coupled with hybrid triple quadrupole time-of-flight mass spectrometry (UHPLC-Q-TOF-MS) combined with an efficient online data acquisition and a multiple data processing method were developed for metabolite identification in vivo (rat plasma, bile, urine and feces) and in vitro (rat liver microsomes and intestinal flora). A total of 51 metabolites in vivo, 60 metabolites in vitro were structurally characterized. The loss of CH_2_, CH_2_O, O, CO, oxidation, methylation, glucuronidation, sulfate conjugation, N-acetylation, hydrogenation, ketone formation, glycine conjugation, glutamine conjugation and glucose conjugation were the main metabolic pathways of eupatorin. This was the first identification of metabolites of eupatorin in vivo and in vitro and it will provide reference and valuable evidence for further development of new pharmaceuticals and pharmacological mechanisms.

## 1. Introduction

**Figure 1:**
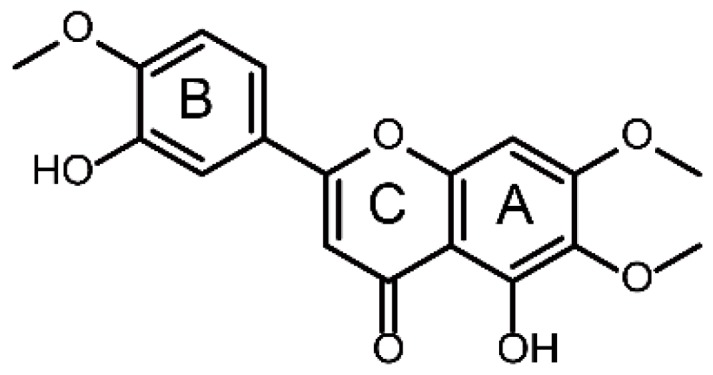
Chemical structure of eupatorin.

Eupatorin (5,3′-di-hydroxy-6,7,4′-tri-methoxy-flavone, Figure 1), belonging to the natural methoxyflavone compound, is widely found in Java tea (*Orthosiphon stamineus*, OS) which is a popular medicinal herb used in traditional Chinese medicine as a diuretic agent and for renal system disorders in Southeast Asia and European countries [1,2,3]. OS has gained a great interest nowadays due to its wide range of pharmacological effects such as antibacterial, antioxidant, hepatoprotection, antidiabetic, anti-hypertension, anti-inflammatory and antiproliferative activities [4,5,6,7,8,9]. Eupatorin, as a major bioactive flavonoid constituent in OS possesses numerous strong biological activities, including anticancer, anti-inflammatory and vasorelaxation activities [10,11,12,13,14,15,16,17]. Its anticancer activities have attracted more and more attention and it was expected to be developed as a cancer chemopreventive and as an adjuvant chemotherapeutic agent. Although there is literature on the qualitative and quantification profile of eupatorin in OS [6], the metabolism study of eupatorin has not been studied to date, which was necessary for the exploration of the biological activity and the clinical therapeutic effect of eupatorin. Thus, an investigation is essential to explore the identification of metabolites of eupatorin for further understanding of its biological activities.

To the best of our knowledge, a series of biotransformations will occur when drugs are orally taken into the body, there are four aspects of pharmacological consequences in these biotransformation processes: (1) Transforming into inactive substances; (2) transforming the drug with no pharmacological activity into active metabolites; (3) changing the types of pharmacological actions of drugs; (4) and producing toxic substances [18]. Therefore, it is extremely crucial to study the metabolism of drugs in vivo to make sure of safety of use. In addition, as the main metabolic organ of the human body, the liver is rich in enzymes, especially cytochrome P450 enzymes, which are closely related to the biological transformation of drugs [19]. Furthermore, the gastrointestinal tract is also a vital place for drug metabolism, and its intestinal flora have a significant impact on drug absorption, metabolism and toxicology [20,21]. Hence, in this paper, mass spectrometry was employed to investigate the metabolism of eupatorin in rats, liver microsomes and intestinal flora, in order to characterize the metabolites and structural information of the products, which will lay a foundation for further studies on the safety and efficacy of metabolites and will provide greater possibilities for the development of new drugs.

With the development of technology, a quadrupole time-of-flight mass spectrometry has been widely used as a reliable analytical technique to detect metabolites due to its advantages of high resolution, high sensitivity, high-efficiency separation and accurate quality measurement [22,23]. In this study, high-sensitivity ultra-high-performance liquid chromatography coupled with hybrid triple quadrupole time-of-flight mass spectrometry (UPLC-Q-TOF-MS) full scan mode, electrospray ionization (ESI) source negative ion mode monitoring combined with multiple mass loss (MMDF) and dynamic background subtraction (DBS) were employed to collect data online. Correspondingly, multiple data processing methods were applied by using PeakView 2.0 and MetabolitePilot 2.0.4 software developed by AB SCIEX company, including a variety of data handing functions such as the extraction of ion chromatograms (XIC), mass defect filter (MDF), product ion filter (PIF) and neutral loss filtering (NLF), which provided accurate secondary mass spectral information [24]. Based on the above methods, the metabolic pathways of eupatorin were explored and summarized for the first time and 51 metabolites in vivo and 60 metabolites in vitro were finally identified. These metabolic studies are important parts of drug discovery and development and can also provide a basis for further pharmacological research.

## 2. Results and Discussion

### 2.1. Analytical Strategy

In this study, UHPLC-Q-TOF-MS/MS combined with an online data acquisition and multifarious processing methods was adopted to systematically identify the metabolites of eupatorin in vivo and in vitro.

The workflow of the analytic procedure was segmented into three steps. First, an online full-scan data acquisition was performed based on the MMDF and DBS to collect data online and to capture all potential metabolites. Next, a multiple data processing method was employed by using PeakView 2.0 and MetabolitePilot 2.0.4 software, which contained many data-processing tools such as XIC, MDF, PIF and NIF, these provided accurate MS/MS information to determine the metabolites of eupatorin. Finally, plenty of metabolites were identified according to accurate mass datasets, specific secondary mass spectrometry information and so on. With regard to the isomers of metabolites, Clog P values calculated by ChemDraw 14.0 were used to further distinguish them. Generally speaking, the larger the Clog P value, the longer the retention time will be in the reversed-phase chromatography system [25,26,27].

### 2.2. Mass Fragmentation Behavior of Eupatorin

**Figure 2:**
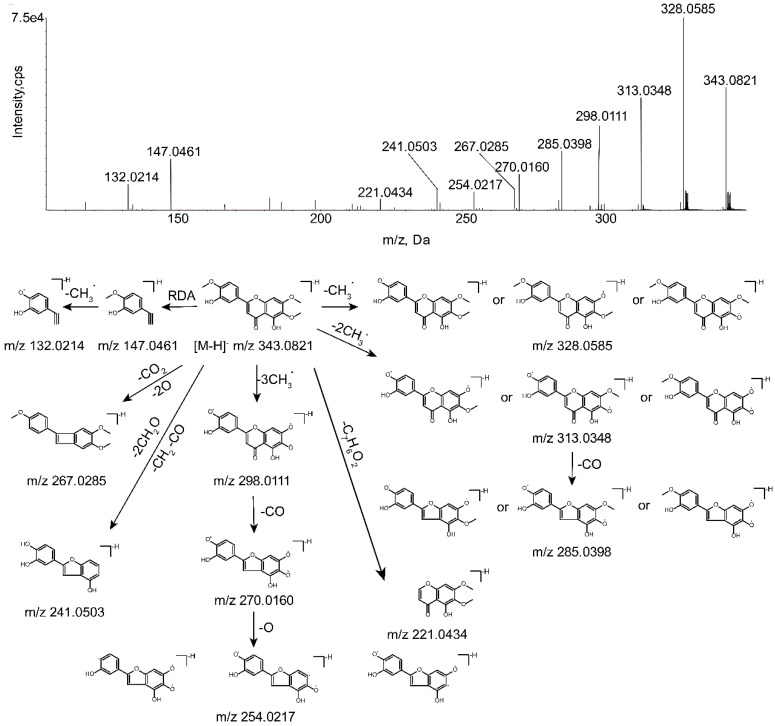
MS/MS spectrum of eupatorin and its predominant fragmentation pathways.

In order to identify the metabolites of eupatorin, it is of significance to understand the pyrolysis of parent drug (M0). The chromatographic and mass spectrometric behaviors of eupatorin were explored in the negative ESI scan mode by UHPLC-Q-TOF-MS. Eupatorin (C_18_H_16_O_7_) was eluted at 12.22 min and yielded at 343.0821 [M-H]^−^. The characteristic fragment ions of M0 at *m*/*z* 328.0585, 313.0348, 298.0111, 285.0398, 270.0160, 267.0285, 254.0217, 241.0503, 221.0434, 147.0461, 132.0214 were detected according to the MS/MS spectrum. Fragment ions at *m*/*z* 328.0585, 313.0348, 298.0111, 270.0160 and 254.0217 were generated by M0 through losing CH_3_, CH_3_, CH_3_, CO and O continuously. The ion at *m*/*z* 343.0821 yielded other representative fragment ions at *m*/*z* 267.0285, 241.0503 and 221.0434 by loss of CO_2_ and 2O, C_4_H_6_O_3_, C_7_H_6_O_2_, respectively. The product ion at *m*/*z* 285.0398 was created by dropping CO from the ion at *m*/*z* 313.0348. Last but not the least, the conspicuous product ion at *m*/*z* 147.0461 was formed because of the Retro-Diels-Alder (RDA) reaction in ring C of the flavonoid, which gained the ion at *m*/*z* 132.0214 by loss of CH_3_ [28]. The MS/MS spectrum and the fragmentation pathways of eupatorin are shown in Figure 2.

### 2.3. Identification of Metabolites in Vivo and in Vitro

Metabolites M1, M2 and M3 (C_17_H_14_O_7_) were isomers with the deprotonated molecular ions [M-H]^−^ at *m*/*z* 329.0660, 329.0668 and 329.0662, which were 14 Da (CH_2_) lower than that of M0. They were eluted at 9.93 min, 10.27 min and 10.79 min, respectively. In the MS/MS spectrum, product ions at *m*/*z* 314.0427, 313.0384, 299.0188 and 285.0371 were formed after losing CH_3_, O, 2CH_3_ and CO_2_, respectively. The prominent fragment ion at *m*/*z* 133.0287 created after the RDA reaction was 14 Da lower than the ion *m*/*z* 147.0461 of the parent drug, suggested that CH_2_ was lost at the methoxy group at 4′position. At the same time, the fragment ions at *m*/*z* 207.7129 and 207.7166 were 14 Da lower than that of M0, which showed that the loss of CH_2_ occurred at the methoxy group at 6 or 7 position of A ring. Additionally, the Clog P values of M1, M2 and M3 were 2.26422, 2.26434, 2.51422, respectively. Therefore, M1–M3 were illustrated according to the above information.

Metabolites M4 and M5 (C_16_H_12_O_7_) were eluted at 7.26 and 8.50 min, with the deprotonated molecular ions [M-H]^−^ at *m*/*z* 315.0500 and 315.0504, 28 Da (C_2_H_4_) lower than that of the parent drug, which indicated that it lost 2CH_2_. Fragment ions at *m*/*z* 300.0279 and 297.1740 were generated by loss of CH_3_ and H_2_O, respectively. The product ion at *m*/*z* 269.1760 was obtained through dropping CO from the ion at *m*/*z* 297.1740. According to the dominant fragment ion at *m*/*z* 133.0270 gained by the RDA reaction, loss of CH_2_ and CH_2_ occurred at the position of 4′, 6 or 4′, 7. In addition, the distinctive ion at *m*/*z* 147.0821 was similar with that of the parent drug, which implied that the reaction occurred at the position of 6 and 7.

Metabolite M6 (C_17_H_14_O_6_) was obtained with a peak at *m*/*z* 313.0713 in the UPLC system, which was eluted at 13.86 min, 30 Da (CH_2_O) lower than that of eupatorin. Prominent fragment ions at *m*/*z* 298.0483 and 283.0250 were created by dropping CH_3_ and CH_3_ successively. In addition, the characteristic fragment ions at *m*/*z* 117.0364 was produced by RDA reaction, which was 30 Da lower than that of M0, showing that loss of CH_2_O occurred at the position of 4′. Similarly, the product ion at *m*/*z* 147.0078 was consistent with M0, indicating that loss of CH_2_O occurred at the position of 6 or 7. Thus, it was speculated that it may have three missing CH_2_O sites.

Metabolite M7 (C_16_H_12_O_6_) was detected at 10.10 min and exhibited the molecular ion [M-H]^−^ at *m*/*z* 299.0562, which was 44 Da lower than that of M0. Based on the information of chemical elements and software provided, it indicated that M7 lost CH_2_O and CH_2_. Crucial fragment ions at *m*/*z* 284.0326 and 251.1281 were obtained by loss of CH_3_ and 3O from M7, respectively. Furthermore, M7 had common fragment ion at *m*/*z* 146.9687 with that of the parent drug, it is equally important that the noteworthy fragment ion at *m*/*z* 281.1787 was generated by loss of H_2_O from M7, which implied that loss of CH_2_O and CH_2_ occurred at the position of 7 or 6, respectively. Hence, it was identified.

Metabolite M8 (C_16_H_12_O_5_) was eluted at 13.60 min, which displayed deprotonated molecular ion [M-H]^−^ at *m*/*z* 283.0614, 60 Da (C_2_H_4_O_2_) lower than that of the parent drug. Fragment ions at *m*/*z* 268.0379 and 240.0428 were produced by dropping CH_3_ and CO continuously from *m*/*z* 283.0614. In addition, the dominant fragment ion at *m*/*z* 146.9655 was consistent with that of the parent drug, while the diagnostic fragment ion at *m*/*z* 161.0025 was 60 Da lower than 221.0434 of M0, these suggested that loss of CH_2_O and CH_2_O reaction happened at C-6 and C-7 of A ring. So, the structure of M8 could be inferred.

Metabolites M9 and M10 (C_18_H_16_O_6_) appeared as deprotonated molecular ions [M-H]^−^ at *m*/*z* 327.0882 and 327.0872, together with the retention time of 4.98 min and 7.47 min, respectively, which were 16 Da lower than M0, suggesting they lacked one oxygen atom compared with the parent. The MS/MS spectra showed the fragment ions at *m*/*z* 309.0800, 299.0957 and 281.2489, which were created by loss of O, CO and C_2_H_6_O, respectively. In addition, M9 had common fragment ion at *m*/*z* 146.9380 with that of the parent drug, and meanwhile the characteristic fragment ion at *m*/*z* 205.0025 was 16 Da lower than 221.0434 of M0, which implied that loss of O occurred at C-5 of A ring. Nevertheless, the ion at *m*/*z* 130.9716 gained after the RDA cleavage was 16 Da lower than that of the parent drug, showing that loss of O occurred at C-4′ of B ring. Therefore, the structures of metabolites M9 and M10 were determined. Moreover, they were also validated with the Clog P values of M9 and M10 which were 2.45814 and 3.44497, respectively.

Metabolite M11 (C_18_H_16_O_5_) was turned up in the chromatogram at 9.55 min with the deprotonated molecular ion at *m*/*z* 311.0930 [M-H]^−^ and was 32 Da less than that of M0, suggesting that the loss of two oxygen atoms reaction took place. A series of diagnostic product ions at *m*/*z* 250.9816, 204.9868 and 130.9658 were yielded by loss of C_2_H_4_O_2_, C_7_H_6_O and RDA reaction. In addition, the product ion at *m*/*z* 174.9556 was obtained through dropping CH_2_O from the ion at *m*/*z* 204.9868. According to the above characteristic fragment ions and analysis, loss of O and O occurred at C-5 and C-3′.

Metabolite M12 (C_17_H_14_O_5_), the deprotonated molecular ion of *m*/*z* 297.0768 was observed at the retention time of 7.33 min and was 46 Da lower than that of eupatorin. According to its secondary mass spectrum and the information software provided, implying that M12 lost O and CH_2_O. Fragment ions at *m*/*z* 267.1016, 253.0865, 175.0394 and 147.0452 were produced by loss of CH_2_O, CO_2_, C_7_H_6_O_2_ and RDA reaction. It was important that the typical ion at *m*/*z* 147.0452 was similar with the fragment ion at *m*/*z* 147.0461 of the parent drug, together with the dominant fragment ion at *m*/*z* 175.0394, 46 Da lower than that of M0, all of which indicated that the reaction was likely to occur in the A ring. Above all, loss of O happened at the hydroxyl group at the 5 position, while loss of CH_2_O occurred at the methoxy group at 6 or 7 position.

Metabolite M13 (C_17_H_16_O_6_) exhibited a sharp peak at an elution time of 12.74 min in the XIC with a deprotonated ion at *m*/*z* 315.0862 and it was 28 Da (CO) less than eupatorin. Product ions at *m*/*z* 300.0633, 285.0401 and 270.0144 were formed after dropping CH_3_ continuously. In addition, the MS^2^ spectrum of M13 presented other vital fragment ions at *m*/*z* 193.0503 and 147.0445 by losing C_7_H_6_O_2_ and undergoing RDA reaction.

Metabolites M14, M15, M16 and M17 (C_18_H_16_O_8_): Four chromatographic peaks were eluted at 10.01 min, 10.50 min, 11.47 min and 12.23 min with deprotonated molecular ions [M-H]^−^ at *m*/*z* 359.0772, 359.0768, 359.0767 and 359.0767, which were 16 Da (O) higher than that of eupatorin. Characteristic ions at *m*/*z* 344.0542, 329.0304, and 314.0064 were obtained by loss of CH_3_ successively. Furthermore, noteworthy fragment ions at *m*/*z* 221.0098 and 163.0368 were produced by loss of C_7_H_6_O_3_ and RDA reaction. The ion at *m*/*z* 163.0368 was 16 Da (O) larger than *m*/*z* 147.0461, showing that oxidation occurred at C-2′, C-5′ or C-6′ of B ring. However, the prominent ion at *m*/*z* 147.0130 was similar with the fragment ion at *m*/*z* 147.0461 of the parent drug, indicating that the reaction happened at the position of 8 in the A ring. The Clog P values of M14-M17 were 1.79518, 1.84518, 1.86518 and 1.87123, respectively. Thus, M14-M17 were characterized by comparing the different values of Clog P.

Metabolite M18 (C_18_H_16_O_9_), the deprotonated molecular ion of *m*/*z* 375.0709 was observed at the retention time of 9.90 min, which was 32 Da (2O) higher than that of eupatorin. A series of product ions at *m*/*z* 329.0669, 221.1216 and 178.9947 were detected by loss of CH_2_O_2_, C_7_H_6_O_4_ and RDA reaction in its secondary mass spectrum. Product ions at *m*/*z* 314.0434 and 299.0191 were produced by losing CH_3_ and CH_3_ continuously from the ion at *m*/*z* 329.0669. What’s more, the key fragment ions at *m*/*z* 178.9947 was 32 Da higher than 147.0461 of eupatorin, implying that di-oxidation reaction occurred in the B ring, then M18 was identified.

Metabolite M19 (C_18_H_16_O_10_) was detected at 12.26 min and showed the deprotonated molecular ion [M-H]^−^ at *m*/*z* 391.0673, 48 Da (3O) higher than that of the parent drug, which contained the fragment ions at *m*/*z* 345.0869, 330.0636 and 315.0393 by loss of CH_2_O_2_, CH_3_ and CH_3_ continuously. More importantly, distinctive fragment ions at *m*/*z* 221.0399 and 195.0289 were created by loss of C_7_H_6_O_5_ and RDA reaction. The pivotal fragment ions at *m*/*z* 195.0289 was 48 Da higher than 147.0461 of the parent drug, suggesting that tri-oxidation happened at C-2′, C-5′ and C-6′ of B ring. Hence, M19 was recognized.

Metabolites M20 and M21 (C_17_H_14_O_8_) were eluted at 9.43 min and 10.29 min, with the deprotonated molecular ions [M-H]^−^ at *m*/*z* 345.0605 and 345.0606 and were increased by 2 Da compared with M0, indicating that it carried out demethylation and oxidation reaction. The representative secondary fragment ions at *m*/*z* 330.0384, 301.0719, 221.0028, 125.0311 and 149.0234 generated by the loss of CH_3_, CO_2_, C_6_H_4_O_3_, C_11_H_8_O_5_ and RDA reaction implied that demethylation and oxidation occurred in ring B. Furthermore, the Clog P values of M20 and M21 were 1.5017 and 1.59734, respectively, so their structures were identified.

Metabolites M22 and M23 (C_19_H_18_O_7_) were obtained in the extracted chromatogram at *m*/*z* 357.0972 and 357.0969 with the retention time of 10.02 min and 12.86 min, which were 14 Da (CH_2_) higher than that of eupatorin. The diagnostic fragment ions at *m*/*z* 342.0740, 327.0503, 312.0266 and 297.0033 were attributed to the loss of CH_3_ successively. In addition, because of the prominent fragment ions at *m*/*z* 235.0434 and 147.0433 obtained after RDA reaction, it was proposed that methylation happened at hydroxyl group at 5 position. Nevertheless, the fragment ion at *m*/*z* 161.0269 was 14 Da higher than 147.0461 of eupatorin, indicating that it occurred at C-3′ of B ring. Furthermore, the Clog P values of M22 and M23 were 2.06632 and 3.18323, respectively, so they were verified.

Metabolites M24 and M25 (C_19_H_18_O_6_) were eluted at 7.15 min and 8.79 min, respectively. They had the deprotonated molecular ions [M-H]^−^ at *m*/*z* 341.1025 and 341.1027, which were 2 Da lower than that of eupatorin, presumably they occurred a loss of O and a methylation reaction. The distinctive fragment ion at *m*/*z* 130.9906 was 16 Da (O) lower than 147.0461 of M0, along with fragment ions at *m*/*z* 235.0607 and 107.0440 produced by loss of C_7_H_6_O and C_12_H_10_O_5_, implying that the loss of O occurred at the hydroxyl group at C-3′, while methylation happened at the hydroxyl group at C-5. Similarly, according to the representative fragment ion at *m*/*z* 161.0595, 14 Da (CH_2_) lower than 147.0461 of M0 and the diagnostic fragment ions at *m*/*z* 204.9196 and 137.0553 obtained by loss of C_8_H_8_O_2_ and C_11_H_8_O_4_, the loss of O occurred at the hydroxyl group at 5 position, while methylation took place at the hydroxyl group at 3′ position. In addition, they were also validated with the Clog P values of M24 and M25 which were 2.80306 and 2.9313, respectively.

Metabolite M26 (C_15_H_10_O_5_), displayed a peak at 9.89 min, as well as a deprotonated molecular ion [M-H]^−^ at *m*/*z* 269.0459, 14 Da (CH_2_) lower than that of M8, suggesting that demethylation occurred on the basis of M8. The fragment ions at *m*/*z* 253.0124, 241.0500, 225.0555 and 133.0298 were attributed to the loss of O, CO, CO_2_ and RDA reaction, which was 14 Da (CH_2_) lower than that of M0 and M8, implying that demethylation occurred at the methoxy group at 4′ position. Like the M8, the loss of CH_2_O and CH_2_O took place at C-6 and C-7 of A ring.

Metabolite M27 (C_15_H_10_O_6_) was detected at a retention time of 8.45 min with the deprotonated molecular ion [M-H]^−^ at *m*/*z* 285.0402, 14 Da (CH_2_) lower than that of M7, indicating that M27 was demethylated on the basis of M7. Product ions at *m*/*z* 267.0130, 241.0462, 221.0063, 177.0189 and 133.0307 were produced by loss of H_2_O, CO_2_, 4O, C_6_H_4_O_2_ and RDA reaction which was 14 Da (CH_2_) lower than that of M0 and M7, it means demethylation happened at the methoxy group at 4′ position. Similar to M7, loss of CH_2_O and CH_2_ occurred at C-7 and C-6 of A ring, respectively.

Metabolites M28 and M29 (C_24_H_24_O_13_) arose as deprotonated molecular ions [M-H]^−^ at *m*/*z* 519.1140 and 519.1151, together with the retention time of 7.10 min and 8.14 min, respectively, which were 176 Da higher than that of eupatorin, suggesting that glucuronidation was carried out. The key product ion at *m*/*z* 343.0822 was yielded by dropping a glucuronic acid. Moreover, the crucial ion at *m*/*z* 146.9662 was similar to the fragment ion at *m*/*z* 147.0461 and while the fragment ion at *m*/*z* 397.0442 was 176 Da higher than that of the parent drug, indicating that glucuronidation happened at the hydroxyl group at 5 position. Nevertheless, the prominent fragment ions at *m*/*z* 323.0173 was 176 Da larger than 147.0461 of M0, inferring that the reaction occurred at the hydroxyl group at 3′ position. Furthermore, M28 and M29 were also proved by the different Clog P values of −0.494983 and 0.621934, respectively.

Metabolite M30 (C_23_H_22_O_13_) was detected at 7.88 min with the deprotonated molecular ion [M-H]^−^ at *m*/*z* 505.0979, 14 Da lower than that of M28 and M29, which suggested that it occurred glucuronide conjugation and demethylation. The characteristic product ion at *m*/*z* 329.0669 was obtained by losing a glucuronic acid. The distinctive fragment ions at *m*/*z* 285.0735 and 309.0687 which was 162 Da larger than 147.0461 of M0 were attributed to the loss of C_11_H_8_O_5_ and RDA reaction, implying glucuronide conjugation and demethylation occurred at the position of 3′ and 4′, respectively.

Metabolites M31 and M32 (C_18_H_16_O_10_S) appeared as deprotonated molecular ions [M-H]^−^ at *m*/*z* 423.0391 and 423.0387 with the retention time of 8.93 min and 9.20 min. S elemental was found, suggesting that it had been a sulfate bound. The characteristic product ion at *m*/*z* 343.0830 was created by the loss of SO_3_. Remaining ions at *m*/*z* 328.0593, 313.0355, 285.0413 and 147.0037 were similar to the fragment ions of the parent drug, inferring that sulfate conjugation occurred at the hydroxyl group at 5 position. However, the pivotal fragment ion at *m*/*z* 227.0084 was 80 Da higher than 147.0461 of eupatorin, implying sulfate conjugation took place at the hydroxyl group at 3′ position of B ring. In addition, the Clog P values of M31 and M32 were 0.270316 and 1.38723, respectively. So, they were also validated.

Metabolite M33 (C_17_H_14_O_10_S) was eluted at the retention time of 9.01 min on the UPLC system. Its deprotonated molecular ion [M-H]^−^ at *m*/*z* 409.0233 lacked CH_2_ compared with M31 and M32. The representative product ion at *m*/*z* 329.0670 was acquired by dropping SO_3_. In addition, M33 created the dominant fragment ion at *m*/*z* 212.0456 through the RDA reaction, and the product ion at *m*/*z* 132.0208 was formed by the loss of SO_3_ from it. Therefore, it might occur at the methoxy group at 4′ position.

Metabolite M34 (C_18_H_16_O_9_S) was eluted at a retention time of 12.65 min. The MS/MS spectrum of M34 showed the deprotonated molecular ion [M-H]^−^ at *m*/*z* 407.0434, lacked one oxygen atom compared with M31 and M32. The crucial fragment ions at *m*/*z* 327.0826, 301.0034 and 131.0573 were attributed to the loss of SO_3_, C_7_H_6_O and RDA reaction. In addition, the product ion at *m*/*z* 220.9818 was acquired by the loss of SO_3_ from the fragment ions at *m*/*z* 301.0034. Based on the information above, the loss of O and sulfate conjugation might occur at the hydroxyl group at 3′ and 5 position, respectively.

Metabolites M35 and M36 (C_20_H_18_O_8_): Two isomers were simultaneously extracted in the XIC at 13.36 and 13.90 min and were detected at *m*/*z* 385.0917 and 385.0925, respectively. The noteworthy ion at *m*/*z* 343.0846 was yielded by the loss of acetyl. In M36, the diagnostic fragment ion at *m*/*z* 189.0551 generated by RDA reaction, which was 42 Da higher than 147.0461 of eupatorin and the distinctive fragment ion at *m*/*z* 221.0781 indicated that acetylation reaction happened at the hydroxyl group at 3′ position. Likewise, according to the prominent fragment ions at *m*/*z* 263.0551 and 147.0513, the acetylation reaction happened at the hydroxyl group at position 5 of M35. In addition, Clog P values of M35 and M36 were 1.49632 and 2.61323, respectively, which could also support the confirmation of the structures.

Metabolites M37 and M38 (C_20_H_18_O_7_) were observed at 13.02 and 13.90 min in the XIC and were detected at *m*/*z* 369.0987 and 369.0975 in the mass spectra, respectively, which were decreased by 16 Da (O) compared with M35 and M36. The typical fragment ion at *m*/*z* 130.9934, 16 Da lower than 147.0461 of eupatorin, together with the representative fragment ion at *m*/*z* 263.1681, 42 Da higher than that of eupatorin, inferring that loss of O and acetylation reaction occurred at the hydroxyl group at 3′and 5 position, respectively. Similarly, based on the crucial product ions at *m*/*z* 174.9586 and 164.9289, loss of O and acetylation reaction occurred at the hydroxyl group at 5 and 3′ position, respectively. In addition, M37 and M38 were also verified based on their Clog P values of 2.23306 and 2.3613, respectively.

Metabolite M39 (C_19_H_16_O_8_) detected at *m*/*z* 371.0761 and eluted at 11.45 min. In addition, it was 14 Da (CH_2_) smaller than the size of M35 and M36. The characteristic ion at *m*/*z* 329.0680 was yielded by dropping of acetyl. The prominent fragment ion at *m*/*z* 175.0389, 28 Da larger than 147.0461 of eupatorin, implying that the loss of CH_2_ and acetylation reaction took place at the methoxy group at 4′ position of B ring.

Metabolites M40 and M41 (C_19_H_16_O_7_) were detected as deprotonated [M-H]^−^ ion at *m*/*z* 355.0813 and 355.0814, which were eluted at 11.86 min and 13.15 min, 30 Da (CH_2_O) lower than that of M35 and M36. In M40, the diagnostic fragment ion at *m*/*z* 117.0329 produced by RDA reaction was 30 less than 147.0461 of the parent drug, while the fragment ion at *m*/*z* 263.0361 was 42 higher than 221.0434 of eupatorin, indicating that loss of CH_2_O and acetylation reaction occurred at the position of 4′ and 5, respectively. However, in M41, the distinctive fragment ion at *m*/*z* 159.10931 was 12 higher than 147.0461 of eupatorin, the crucial fragment ion at *m*/*z* 221.0027 was similar to fragment ion at *m*/*z* 221.0434, suggesting that the loss of CH_2_O still occurred at the methoxy group at 4′ position while acetylation reaction took place at the hydroxyl group at 3′ position. The respective Clog P values were 1.64857 and 2.87497, so M40 and M41 were ensured.

Metabolite M42 (C_21_H_18_O_8_) with the [M-H]^−^ ion of *m*/*z* 397.0918, which was eluted at 15.21 min, 42 Da higher than that of M40 and M41, speculating that the loss of CH_2_O and di-acetylation of amines took place. The characteristic fragment ion at *m*/*z* 159.0462 created by RDA reaction was 12 larger than 147.0461 of the parent drug, while the product ion at *m*/*z* 263.0559 increased by 42 Da compared with 221.0434 of eupatorin, inferring that loss of CH_2_O occurred at the position of 4′ like M40 and M41, while di-acetylation reaction happened at the hydroxyl group at 5 and 3′ position.

Metabolite M43 (C_20_H_16_O_9_), eluted at 14.14 min, which was detected with the deprotonated molecular ion [M-H]^−^ at *m*/*z* 399.0704, 84 Da higher than that of M4 and M5, implying that di-acetylation reaction happened on the basis of the loss of CH_2_ and CH_2_. The diagnostic fragment ions at *m*/*z* 357.0641 and 315.0523 were attributed to the loss of C_2_H_2_O consecutively. Moreover, according to the product ions at *m*/*z* 175.0034 and 147.0316 acquired by RDA reaction, it may have three possible metabolites.

Metabolite M44 (C_20_H_16_O_7_) exhibited a sharp peak at an elution time of 15.24 min in the XIC with a deprotonated ion at *m*/*z* 367.0806 and it was 32 Da (2O) lower than M43, suggesting that the loss of CH_2_O and CH_2_O and di-acetylation reaction occurred. The noteworthy fragment ion at *m*/*z* 283.0237 was yielded by dropping of 2C_2_H_2_O. M44 generated the fragment ions at *m*/*z* 189.0633 and 159.0348 after RDA reaction, so the possible structures were inferred according to above MS/MS information.

Metabolites M45, M46, M47 and M48, eluted at 4.57 min, 5.14 min, 5.43 min, 9.69 min, respectively, all exhibited the deprotonated ion at *m*/*z* 329.1028, 329.1029, 329.1029, 329.1029, implying that they were isomers with the molecular formula C_18_H_18_O_6_ and were 2 Da higher than that of M9 and M10, so hydrogenation happened on the basis of the loss of O. In M45, the representative product ion at *m*/*z* 130.9874 obtained by RDA reaction was 16 (O) less than 147.0461 of eupatorin, while the fragment ion at *m*/*z* 223.0900 was twice higher than 221.0434 of eupatorin, indicating that the loss of O and hydrogenation happened at the position of 3′ and 4, respectively. Like M45, according to the crucial product ions at *m*/*z* 147.0535 and 207.0777, 149.0538 and 207.0618, 133.0731 and 223.0742, the structures of M46, M47 and M48 were distinguished by the analysis above. Furthermore, M45, M46, M47 and M48 were also proved by the different Clog P values of 1.71027, 1.7953, 2.28982 and 2.89116, respectively.

Metabolites M49 and M50 (C_18_H_18_O_5_) were observed in the extracted chromatogram at *m*/*z* 313.1080 and 313.1086 with the retention time of 7.61 min and 7.88 min, 2 Da higher than that of M11, while lacked one oxygen atom compared with M45, M46, M47 and M48. In M49, the distinctive product ion at *m*/*z* 130.9677 obtained by the RDA reaction was 16 (O) less than 147.0461 of eupatorin, inferring that hydrogenation happened at the position of 4′. Nevertheless, the characteristic fragment ion at *m*/*z* 133.0654 yielded in M49 by the RDA reaction was 14 less than 147.0461, so hydrogenation happened at the position of 2 and 3. Besides, M49 and M50 were also checked by the different Clog P values of 2.5321 and 3.02662, respectively.

Metabolite M51 (C_17_H_16_O_7_) was obtained with a peak at *m*/*z* 331.0824 in the UPLC system, which was eluted at 10.05 min, 2 Da larger than that of M1, M2 and M3. According to MS/MS spectrum, diagnostic product ions at *m*/*z* 316.0595, 313.1396, 223.1657, 109.0288 and 135.0450 were formed by losing CH_3_, H_2_O, C_6_H_4_O_2_, C_11_H_10_O_5_ and RDA reaction. It’s worth mentioning that the fragment ion at *m*/*z* 135.0450 was 12 less than 147.0461 of eupatorin, so demethylation happened at the methoxy group at 4′ position and hydrogenation occurred at the position of 2 and 3.

Metabolite M52 (C_18_H_20_O_7_) was eluted at the retention time of 12.78 min. Its deprotonated molecular ion [M-H]^−^ at *m*/*z* 347.1140 was increased 4 Da compared with eupatorin, so di-hydrogenation occurred. According to the dominant product ion at *m*/*z* 149.0591 obtained by RDA reaction, 2 Da higher than 147.0461 of eupatorin, and the fragment ion at *m*/*z* 225.0074, 4 Da higher than 221.0434, indicating that di-hydrogenation happened at the position of 2, 3 and 4.

Metabolites M53 and M54 (C_18_H_20_O_6_) were observed in the chromatogram at *m*/*z* 331.1186 and 331.1185 with the retention time of 3.60 min and 4.07 min, respectively, 16 Da (O) lower than that of M52, inferring that the loss of O happened on the basis of di-hydrogenation. The crucial ion at *m*/*z* 133.0325 was generated after the RDA cleavage, which was less than *m*/*z* 147.0461 one oxygen, so the loss of O occurred at the hydroxyl group at 3′ position in M53. However, according to the characteristic product ions at *m*/*z* 149.0680 and 209.0813, the loss of O happened at the hydroxyl group at 5 position while the di-hydrogenation was at the same position as M52. The respective Clog P values were 1.55427 and 1.6393, so M53 and M54 were verified.

Metabolite M55 (C_18_H_20_O_5_) was eluted at 6.36 min possessing the deprotonated molecular ion [M-H]^−^ at *m*/*z* 315.1214, which was 16 Da (O) lower than that of M53, M54 and 4 Da higher than that of M11. Based on the previous analysis of M11, M53 and M54, the structure of M55 can be inferred.

Metabolites M56, M57 and M58 (C_17_H_18_O_7_): Three chromatographic peaks were eluted at 10.18 min, 10.24 min and 10.80 min with deprotonated molecular ions [M-H]^−^ at *m*/*z* 333.0972, 333.0982 and 333.0979, which were 4 Da larger than the size of M1-M3 and 14 Da (CH_2_) higher than that of M52. According to the prominent product ions at *m*/*z* 149.0642 and 135.1164, together with the information of M1–M3 and M52, the structures of M56-M58 could be identified. In addition, M56, M57 and M58 were also ensured by the different Clog P values of 0.324751, 0.371274 and 0.644751, respectively.

Metabolite M59 (C_16_H_16_O_7_) was observed with a peak at *m*/*z* 319.0815 in the chromatogram, which was eluted at 8.47 min, 4 Da larger than that of M3 and M4. According to the MS/MS information, the typical fragment ions at *m*/*z* 301.0701, 211.0353, 197.0452, 149.0269 and 135.0443 were created by loss of H_2_O, C_6_H_4_O_2_, C_7_H_6_O_2_ and RDA reaction, so there were three possible metabolites of M59.

Metabolites M60 and M61 (C_16_H_16_O_5_) appeared as deprotonated molecular ions [M-H]^−^ at *m*/*z* 287.0923 and 287.0927, together with the retention time of 9.97 min and 11.07 min, respectively, which were 4 Da higher than M8, indicating that M60 and M61 might undergo the loss of CH_2_O and CH_2_O reaction followed by di-hydrogenation. In the secondary mass spectrum of M61, it obtained the fragment ions at *m*/*z* 272.0695, 241.2138, 165.0166, 123.0117 and 149.0683 yielded by dropping of CH_3_, CH_2_O_2_, C_7_H_6_O_2_, C_9_H_8_O_3_ and RDA cleavage, so the loss of CH_2_O and CH_2_O reaction happened at the positions of 6 and 7 while di-hydrogenation happened at the positions of 2, 3 and 4. However, the characteristic fragment ions at *m*/*z* 195.0653, 93.0325 and 119.0500 produced by the loss of C_6_H_4_O, C_10_H_10_O_4_ and RDA cleavage. So, there were two positions (4′, 7 or 4′, 6) to have lost CH_2_O. Finally, the sizes of different Clog P values were combined to determine the structure of M60.

Metabolite M62 (C_17_H_18_O_5_) was eluted at 7.37 min, which displayed deprotonated molecular ion [M-H]^−^ at *m*/*z* 301.1078, 4 Da larger the size of M12. In M62, the characteristic fragment ion at *m*/*z* 149.0605 was twice higher than 147.0461 of eupatorin, while, the prominent fragment ion at *m*/*z* 179.0711 was 42 times lower than fragment ion at *m*/*z* 221.0434, so the loss of O, CH_2_O and di-hydrogenation occurred at the same positions as M12 and M52, respectively.

Metabolite M63 (C_15_H_10_O_4_), the deprotonated molecular ion of *m*/*z* 253.0512 was observed at the retention time of 7.81 min, which was 30 Da (CH_2_O) lower than that of M8. M63 comprised the typical fragment ions at *m*/*z* 225.0558, 209.0606, 161.0249 and 117.0351 by dropping of CO, CO_2_, C_6_H_4_O and RDA cleavage. And the fragment ion at *m*/*z* 101.0246 arose by loss of O from the ion at *m*/*z* 117.0351, so the structure was inferred.

Metabolites M64, M65 and M66 (C_18_H_14_O_8_) were the isomeric metabolites with the deprotonated [M-H]^−^ ions at *m*/*z* 357.0607, 357.0609 and 357.0610, 14 Da higher than that of eupatorin, which were eluted at 10.79 min, 10.81 min and 12.99 min, respectively, suggesting that ketone formation reaction occurred. Several conspicuous ions at *m*/*z* 342.0374, 327.0132, 313.0304, 221.0224, 235.0267, 161.0174 and 147.0012 all appeared in the secondary mass spectra after the loss of CH_3_, CH_3_, CO_2_, C_7_H_4_O_3_, C_7_H_6_O_2_ and RDA reaction. Moreover, the Clog P values of M64, M65 and M66 were 2.17223, 2.27223 and 2.52223, respectively. In consequence, the structures of M64, M65 and M66 were distinguished according to the above information.

Metabolite M67 (C_18_H_18_O_9_) was eluted at 12.29 min and showed the deprotonated molecular ion [M-H]^−^ at *m*/*z* 377.0875, 18 Da higher than that of M14-M17, implying that M67 might undergo oxidation followed by internal hydrolysis. In the MS/MS spectrum of M67, the representative product ion at *m*/*z* 181.0136 tested after RDA reaction was 34 larger than 147.0461 of eupatorin, while the fragment ion at *m*/*z* 239.0435 was 18 times higher than that of eupatorin, so internal hydrolysis happened at C-2 and C-3 and oxidation is most likely to occur at C-5′ [29].

Metabolite M68 (C_19_H_17_NO_8_) was observed with a peak at *m*/*z* 386.0865 in the UPLC system, which was eluted at 10.00 min, 57 Da higher than that of M1-M3. The fragment ion at *m*/*z* 329.0662 was acquired, corresponding to the loss of glycine. Additionally, the conspicuous fragment ions at *m*/*z* 264.1192 and 147.0973 were yielded through the loss of C_7_H_6_O_2_ and RDA reaction, implying that the loss of CH_2_ and glycine conjugation were connected to A ring. Hence, there were two possible metabolites of M68.

Metabolite M69 (C_19_H_17_NO_6_) was detected at 6.09 min, which presented an accurate deprotonated ion [M-H]^−^ at *m*/*z* 354.0992, 57 Da higher than that of M12, indicating that M69 might experience the loss of O and CH_2_O reaction followed by glycine conjugation. A sequence of crucial fragment ions at *m*/*z* 324.2008, 250.9077, 174.9553 and 204.0387 were produced by the loss of 2CH_3_, C_3_H_5_NO_3_, C_9_H_9_NO_3_ and RDA reaction, while the characteristic fragment ion at *m*/*z* 204.0387 was 57 higher than 147.0461 of eupatorin, inferring that glycine conjugation was connected to B ring and the loss of O and CH_2_O reaction was at the same position as M12.

Metabolite M70 (C_22_H_22_N_2_O_8_) exhibited a sharp peak at an elution time of 5.07 min in the XIC with a deprotonated ion at *m*/*z* 441.1302. The characteristic fragment ion at *m*/*z* 312.8496 was observed, corresponding to the loss of glutamine [24]. Furthermore, A strong ion at *m*/*z* 245.1021 appeared in the secondary mass spectrum of M70 after the RDA reaction, and was 98 higher than 147.0461 of eupatorin, which created the prominent fragment ion at *m*/*z* 117.2304 by dropping of glutamine, suggesting that the loss of CH_2_O occurred at the methoxy group at 4′ position while glutamine conjugation took place at the hydroxyl group at 3′ position.

Metabolite M71 (C_22_H_22_O_12_), displayed a peak at 5.49 min, as well as a deprotonated molecular ion [M-H]^−^ at *m*/*z* 477.1036. The predominated fragment ion at *m*/*z* 315.6277 was attributed to the loss of glucose. In addition, the distinctive fragment ion at *m*/*z* 146.9654 resulted from RDA reaction was consistent with *m*/*z* 147.0461 of eupatorin, further noteworthy MS/MS fragment ions at *m*/*z* 355.0661 and 192.9548 were yielded corresponding to the consecutive loss of C_7_H_6_O_2_ and glucose. Thus, the loss of CH_2_ and CH_2_ took place at the methoxy group at 6 and 7 position, while glucose conjugation happened at the hydroxyl group at 5 position.

**Figure 3:**
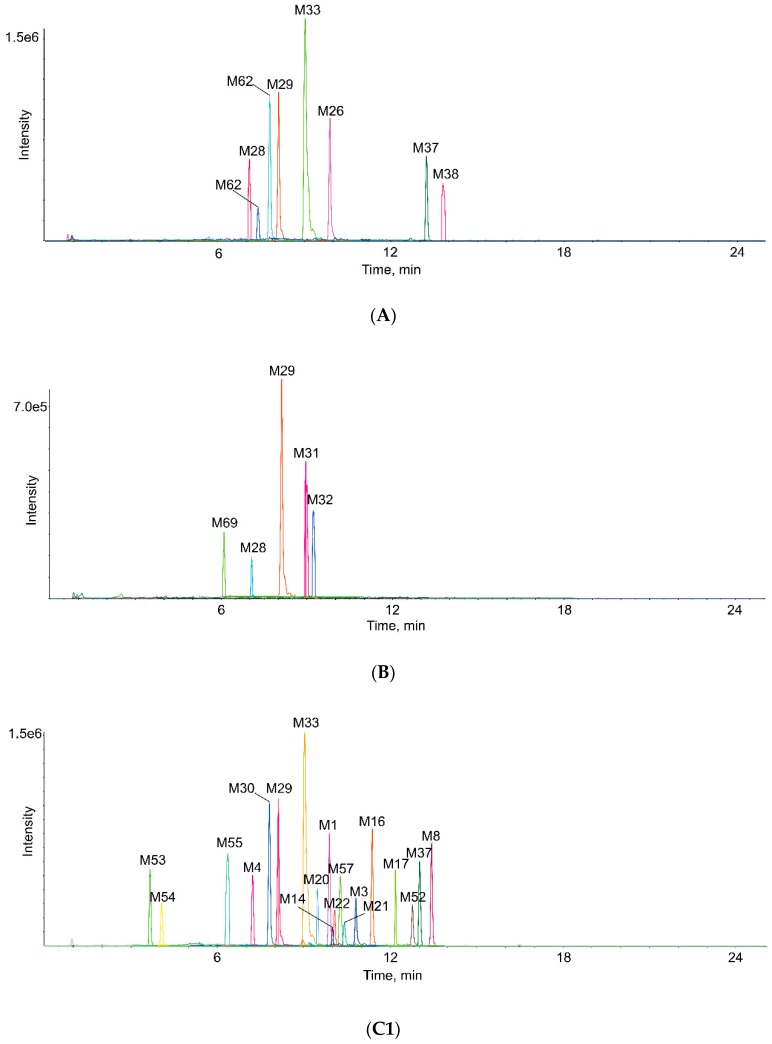

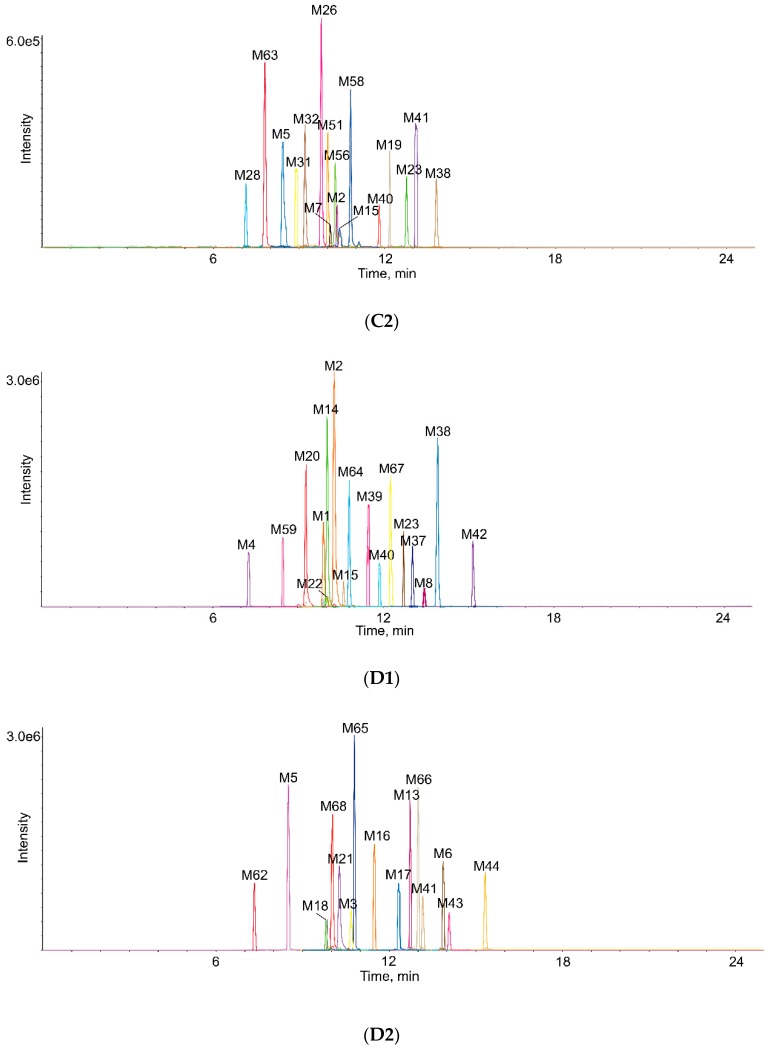

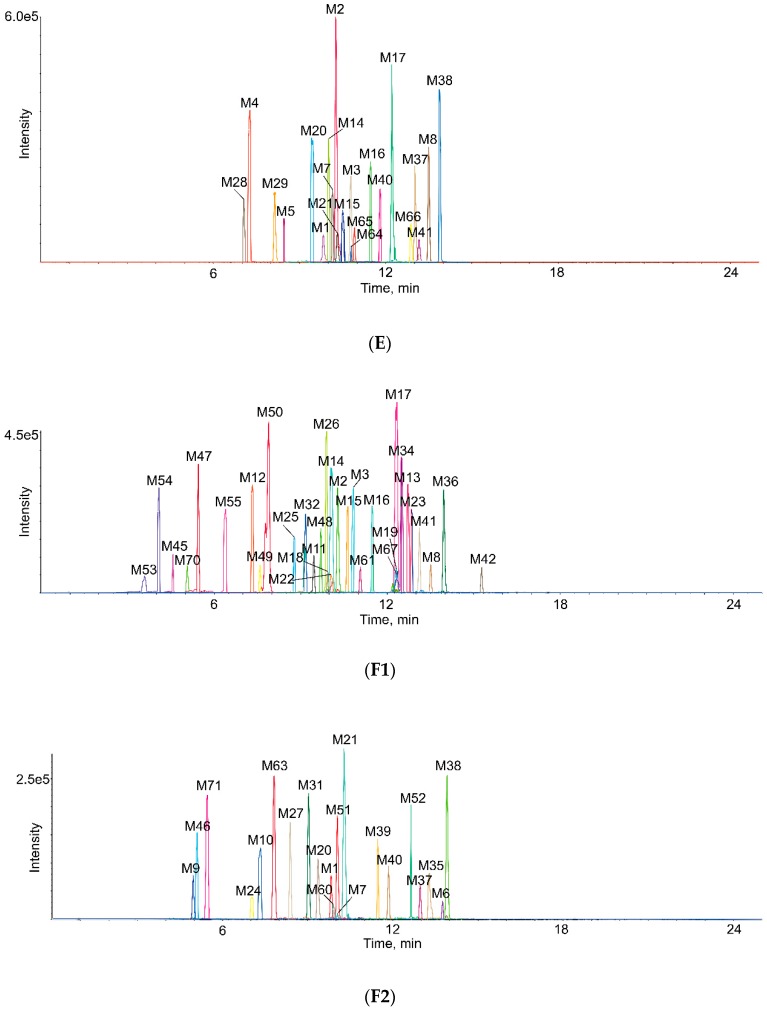
Extracted ion chromatograms of all metabolites of eupatorin in vivo and in vitro (**A**—in rat plasma sample, **B**—in rat bile sample, **C1**,**C2** in rat urine sample, **D1**,**D2** in rat feces sample, **E**—in rat liver microsomes, **F1**,**F2** in rat intestinal flora).

The detected metabolites are listed in Table 1. Moreover, their XICs are exhibited in Figure 3.

### 2.4. Metabolic Pathways of Eupatorin

**Figure 4:**
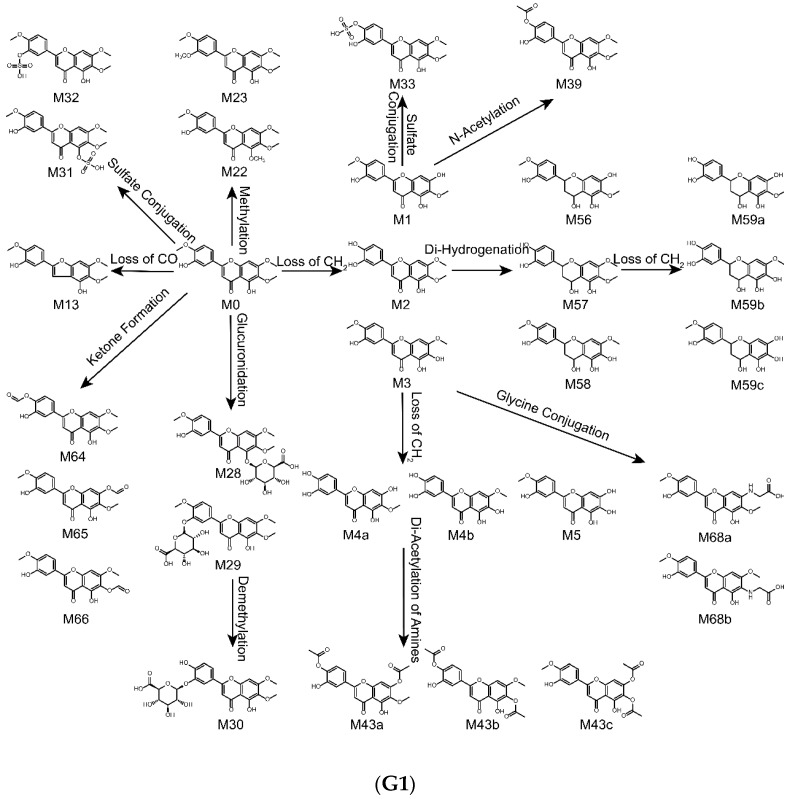

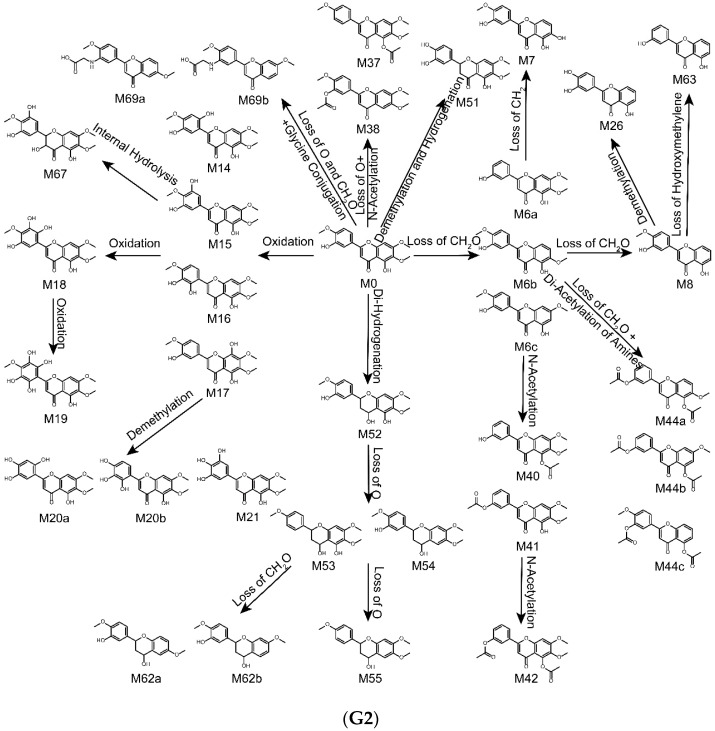

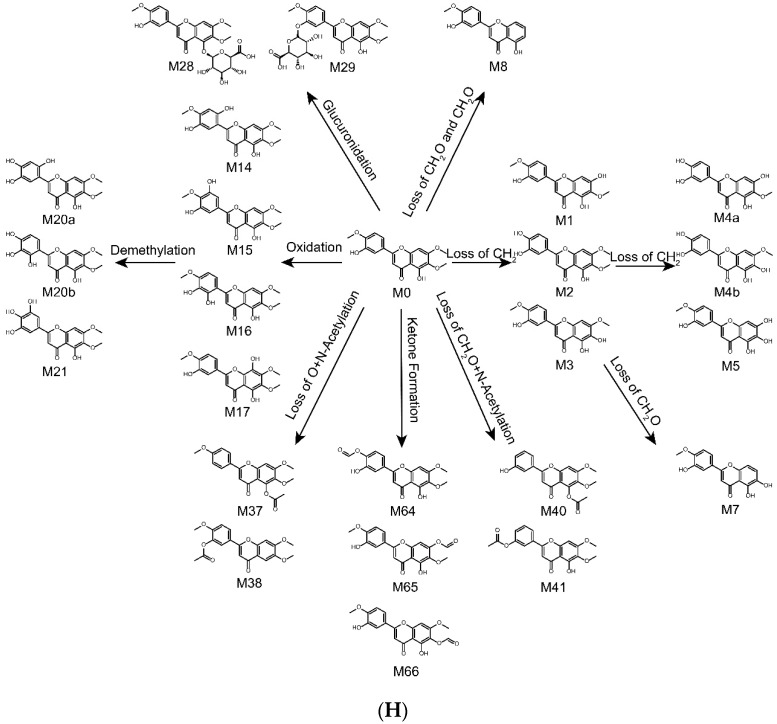

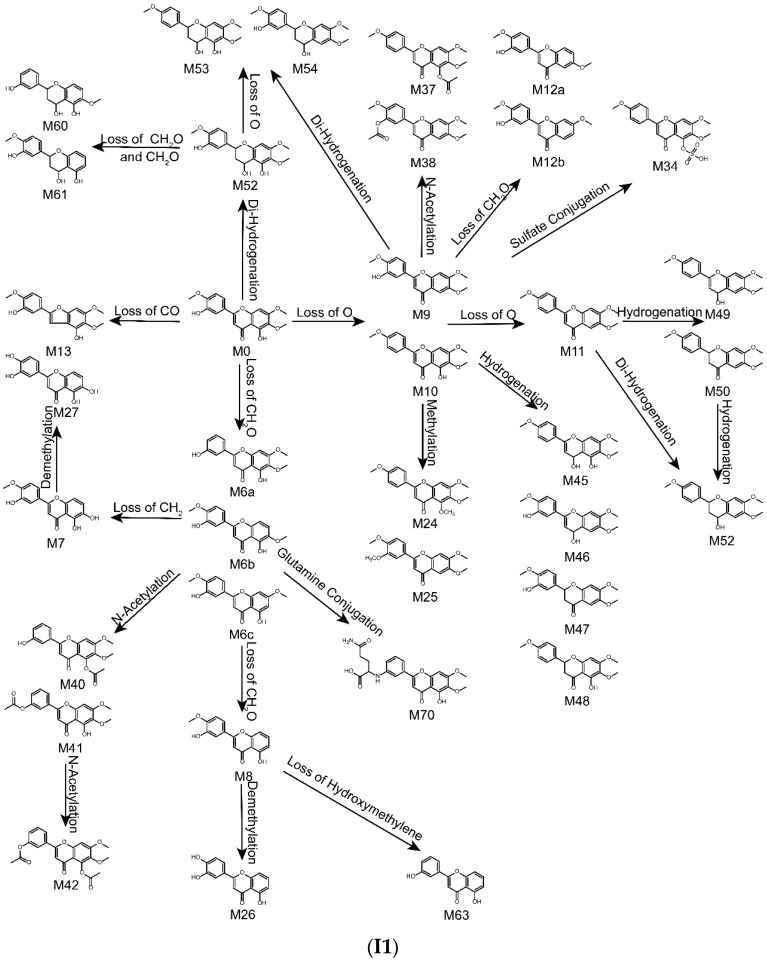

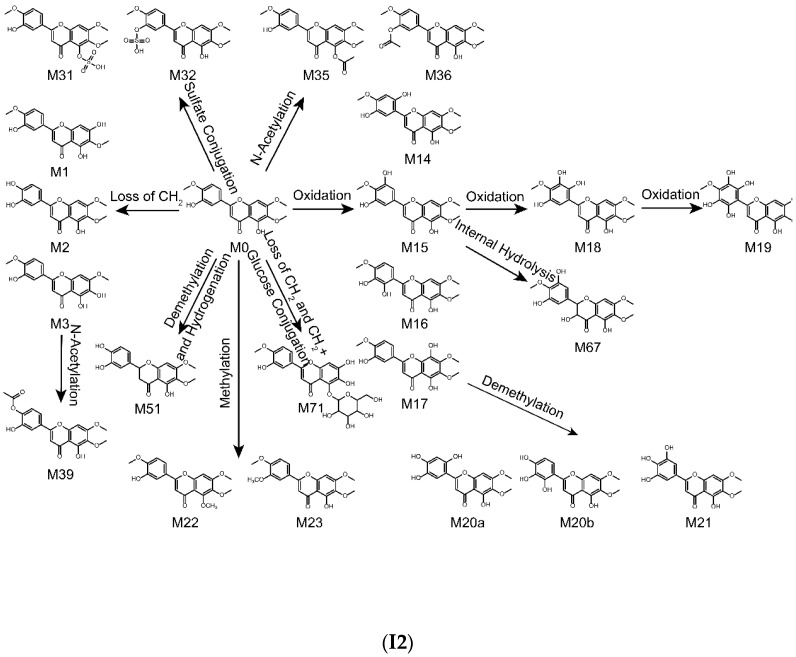
Metabolic profile and proposed metabolic pathways of eupatorin in vivo and in vitro (**G1**,**G2** in vivo, **H** in rat liver microsomes, **I1**,**I2** in rat intestinal flora).

The metabolites of eupatorin in rats after oral administration, in liver microsomes and intestinal flora through incubation was identified in this study. As a result, a total of 51 metabolites in vivo were detected, including 8 metabolites in plasma, 5 metabolites in bile, 36 metabolites in urine and 32 metabolites in feces. Meanwhile, 60 metabolites in vitro were observed, including 22 metabolites in liver microsomes and 53 metabolites in intestinal flora. The proposed metabolic pathways of eupatorin in vivo, in rat liver microsomes and in rat intestinal flora were shown in Figure 4. It is worth mentioning that the loss of CH_2_, CH_2_O, O, oxidation, glucuronidation and ketone formation was the primary metabolic step that produced further reactions such as sulfate conjugation, hydrogenation, N-acetylation, methylation, demethylation, internal hydrolysis, glycine conjugation, glutamine conjugation and glucose conjugation. Moreover, all metabolic changes above had taken place in vivo and in vitro. However, glycine conjugation was just present in vivo, while glutamine conjugation and glucose conjugation merely existed in vitro.

### 2.5. Comparison of Metabolites in Vivo and in Vitro

Drug metabolism plays a significant impact on various fields of pharmaceutical mechanisms as well as drug development and clinical use. In this work, the metabolism of eupatorin in vivo (plasma, bile, urine and feces) and in vitro (rat liver microsomes and intestinal flora) was investigated. In vivo; rat urine and feces possessed high activity for eupatorin metabolism, which were identified as having 36 and 32 metabolites, respectively. Nevertheless, only 8 metabolites were observed in rat plasma and 5 metabolites were detected in rat bile, suggesting that the rat plasma and bile might hold low biotransformation activity [30]. In vitro, 53 metabolites were obtained in rat intestinal flora while 22 metabolites were identified in rat liver microsomes, which implied that most metabolites could be excreted in intestinal flora samples and intestinal tract was more suitable for rapid identification of metabolites of eupatorin in vitro, with enormous catalytic and metabolic capacity which exceeds that of the liver microsomes [24]. Thus, the intestinal tract is considered as an extremely vital organ in the biotransformation of eupatorin.

### 2.6. Metabolite Activity of Eupatorin

It has been reported in the literature that OS was taken as a beverage to improve health and for treatment of kidney disease, bladder inflammation and urethritis [1,2]. As its major active ingredient, eupatorin has also been reported to have meaningful anti-inflammatory activity [15,16]. In this study, the metabolites of eupatorin in urine samples were the largest, which may be related to the therapeutic effects of cystitis, nephritis and urethritis. In addition, many of the metabolites of eupatorin have been studied. For example, M4a namely nepetin, is a natural flavonoid present in different plants. In recent years, accumulating evidence has shown that nepetin exhibits various pharmacological activities, especially potent anti-inflammatory properties, which might be related to the strong anti-inflammatory activity of eupatorin [30,31,32]. Overall, the identification of metabolites of eupatorin provides a basis for new pharmacological studies and these metabolites will be further explored in the future.

## 3. Material and Methods

### 3.1. Chemicals and Materials

Eupatorin (855-96-9, purity > 98.94%) was purchased from Chengdu Desite Co., Ltd. (Chengdu, China). Beta-nicotinamide adenine dinucleotide phosphate (β-NADPH) was purchased from Sigma Chemical (St. Louis, MO, USA). Alamethicin and uridine 5′-diphosphoglucuronic acid trisodium salt (UDPGA) were purchased from BD Biosciences (Woburn, MA, USA). Phosphate buffer saline (PBS) was purchased from Sangon Biotech Co., Ltd. (Shanghai, China). Acetonitrile and methanol were all HPLC grade and were purchased from J.T.-Baker Company (Phillipsburg, NJ, USA). Formic acid (HPLC grade) was provided by Diamond Technology (Dikma Technologies Inc., Lake Forest, CA, USA). Purified water was purchased from Wahaha (Hangzhou Wahaha Group Co., Ltd., Hangzhou, China). L-ascorbic acid, L-cysteine, eurythrol, tryptone and nutrient agar were purchased from Beijing AoBoXing Bio-tech Co., Ltd. (Beijing, China). Sodium carboxymethyl cellulose (CMC-Na), sodium carbonate (Na_2_CO_3_), magnesium chloride (MgCl_2_), potassium dihydrogen phosphate (KH_2_PO_4_), dipotassium phosphate (K_2_HPO_4_), calcium chloride (CaCl_2_), ammonium sulfate ((NH_4_)_2_SO_4_), sodium chloride (NaCl) and magnesium sulfate (MgSO_4_) were obtained from Tianjin Guangfu Technology Development Co., Ltd. (Tianjin, China).

### 3.2. Instruments and Conditions

UHPLC-Q-TOF-MS/MS analysis was performed on a Nexera-X2 UHPLC system (Shimadzu Corp., Kyoto, Japan), which was combined with a triple TOF^TM^ 5600^+^ MS/MS system (AB SCIEX, Concord, Ontario, Canada). The chromatographic separation was achieved on Poroshell 120 EC-C_18_ column (2.1 × 100 mm, 2.7 μm) with a SecurityGuard^®^ UHPLC C18 pre-column (Poroshell).

The mobile phase was composed of 0.1% aqueous formic acid (eluent A) and acetonitrile (eluent B). The gradient elution program was as follows: 10–55% B from 0 to 15 min, 55–95% B from 15 to 20 min, 95–95% B from 20 to 25 min. The column temperature remained at 40 °C. In addition, the injection flow rate and the volume were set at 0.3 mL/min and 3 μL, respectively. Before the next injection, equilibration was performed for 3 min.

Mass spectrometric detection was carried out by a Triple TOF^TM^ 5600 system equipped with Duo-Spray^TM^ ion sources in the negative electrospray ionization (ESI) mode. The following mass spectrometry parameter settings were applied: ion spray voltage (IS), −4.5 kV; the turbo spray temperature, 550 °C; the optimized delustering potential (DP), −60 V; collision energy (CE), −10 eV; and the collision energy spread (CES), 15 eV. Moreover, the nebulizer gas (gas 1), the heater gas (gas 2) and the curtain gas were set to 55, 55 and 35 L/min, respectively.

### 3.3. Metabolism in Vivo

#### 3.3.1. Animals and Drug Administration

Eighteen male Sprague-Dawley (SD) rats (220–220 g, 12–14 weeks old) were purchased from the Experimental Animal Research Center of Hebei Medical University (Certificate No.1811164). The conditions of temperature (22–25 °C), humidity (55–60%) and light (12 h light/dark cycle) were standard for 7 days before being used. All rats were fasted for 12 h but allowed water before the experiments. These rats were divided into six groups randomly with three rats per group. Groups 1, 3 and 5 were the control groups for blank blood, blank bile, blank urine and feces, respectively. Groups 2, 4 and 6 were the drug groups for blood, bile, urine and feces, respectively. Rats in groups 2, 4, 6 were given eupatorin by gavage, which dissolved in a 0.5% CMC-Na solution at a dose of 50 mg/kg. Nevertheless, an equal 0.5% CMC-Na solution without eupatorin was orally given to groups 1, 3, 5. All rat experiments were conducted in accordance with the committee’s guidelines on the Care and Use of Laboratory Animals.

#### 3.3.2. Bio-Sample Collection

The plasma samples collection: About 300 μL–500 μL for each blood sample was gathered from the eye canthus of rat into 1.5 mL heparinized tubes at 0.083, 0.167, 0.25, 0.5, 1, 2, 3, 6, 9, 12 and 24 h after gavage. Every blood sample were centrifuged immediately at 1920 g for 10 min at 4 °C to collect the supernatant. After that all collected plasma samples were combined and stored at −80 °C.

The bile collection: Each rat was injected 20% urethane solution intraperitoneally with 1–2 mL to anesthetize the rats after gavage. Then the rats were performed with bile duct cannulation operation and the bile samples were gathered during 0–1 h, 1–3 h, 3–5 h, 5–8 h, 8–12 h, 12–20 h and 20–24 h with PE-10 tubes (ID = 0.07 cm) [33,34]. Lastly, all bile samples were consolidated and frozen at −80 °C.

The urine and feces collection: The rats were separately housed in metabolic cages with free access to deionized water to collect the urine and feces samples over a 0–72 h period after gavage [35,36]. Finally, all the urine and feces samples were separately mixed, and they were placed at −80 °C before pretreatment was conducted.

#### 3.3.3. Bio-Sample Pretreatment

All biological samples were disposed with two methods: Protein precipitation and liquid-liquid extraction were performed on the combined plasma, bile and urine with three times of methanol and ethyl acetate, respectively. Next, the mixture was vortexed for 5 min and centrifuged at 21,380× *g* for 10 min at 4 °C to obtain the supernatant, which was then collected and dried under nitrogen flow.

Dried and powdered feces samples were severally added to 3-fold methanol and ethyl acetate and then were ultrasonically extracted for 45 min. After centrifugation for 10 min at 21,380× *g*, they were dried under nitrogen gas like the supernatant in plasma, bile and urine samples.

150 μL methanol was added to the residua above with an ultrasonic operation for 15 min, centrifugation at 21,380× *g* for 10 min to gain the supernatant which were ultimately passed through the 0.22 μm millipore filters before injecting into the chromatographic system for further analysis. The control group was handled the same as the drug group.

### 3.4. Metabolism in Vitro by Rat Liver Microsomes

#### 3.4.1. Phase I Metabolism

The typical incubation mixture was carried out in a PBS buffer (pH 7.4) with a final volume of 200 μL, which consisted of liver microsomal protein (1.0 mg/mL), eupatorin (100 μmol/L), MgCl_2_ (3.3 mmol/L), and β-NADPH (1.3 mmol/L) [37]. Preincubation was conducted at 37 °C for 5 min, subsequently NADPH was added to start the reaction. After incubation at 37 °C for 90 min, the reaction was terminated by adding 1 mL of ethyl acetate. Next, vortex and centrifugation for 5 and 10 min, respectively, and then the organic phase was gathered and evaporated under nitrogen gas. 100 μL of acetonitrile was put in the residua and they were eventually passed through the 0.22 μm millipore filters and placed at −20 °C before analysis. Groups contained blank groups incubated without the addition of eupatorin, the control groups incubated without the addition of NADPH and the sample groups, which were implemented in triplicate with the same treatment [38,39].

#### 3.4.2. Phase II Metabolism

The representative incubation mixture was performed in a PBS buffer (pH 7.4) with a final volume of 200 μL, which including liver microsomal protein (1.0 mg/mL), eupatorin (100 μmol/L), MgCl_2_ (3.3 mmol/L), and UDPGA (2 mmol/L). Preincubation was implemented at 37 °C for 20 min, subsequently UDPGA was added to begin the reaction. After incubation at 37 °C for 1 h, the reaction was ceased by adding 200 μL of ice-acetonitrile. Next, vortex and centrifugation for 5 and 10 min, respectively. In addition, the supernatant was passed through the 0.22 μm millipore filter before injecting into the UHPLC-Q-TOF-MS/MS system for analysis. Groups contained blank groups incubated without the addition of eupatorin, the control groups incubated without the addition of UDPGA and the sample groups, which were carried out in triplicate with the same treatment.

### 3.5. Metabolism in Vitro by Rat Intestinal Flora

#### 3.5.1. Preparation of Anaerobic Culture Medium

Solution A: K_2_HPO_4_ (0.78%) 37.5 mL; Solution B: KH_2_PO_4_ (0.47%), NaCl (1.18%), (NH_4_)_2_SO_4_ (1.2%), CaCl_2_ (0.12%) and MgSO_4_ (0.25%) 37.5 mL; Solution C: Na_2_CO_3_ (8%) 50 mL; Solution D: L-ascorbic acid (25%) 2 mL together with L-cysteine 0.5 g, eurythrol 1 g, tryptone 1 g and nutrient agar 1 g, which were all mixed up. Ultrapure water was added to 1 L and then HCl (1 mol/L) was put to adjust the pH of the solution to 7.5–8.0.

#### 3.5.2. Preparation of Intestinal Flora Culture Solution

Fresh intestinal contents (3 g) taken from SD rats were combined with anaerobic culture medium (30 mL) instantly. After stirring with a glass rod, filtered with gauze to obtain the intestinal bacterial liquid.

#### 3.5.3. Sample Preparation

Eupatorin (1 mg/ mL,100 μL) was added to intestinal flora culture medium (1 mL), which was then filled with nitrogen without oxygen. The reactions were terminated by adding 3 volumes of methanol after incubation for 12 h. Next, the mixtures were vortexed for 5 min and centrifuged for 10 min at 21,380 g. Subsequently, the organic phases were collected and evaporated under nitrogen gas, and 100 μL of methanol was added to the residua, vortexed and centrifuged again for 5 and 10 min, respectively. Before analysis, the supernatant was passed through the 0.22 μm millipore filter. Blank groups were incubated without eupatorin, meanwhile the control groups were incubated not in intestinal flora culture solution but in anaerobic culture medium, but others were the same.

## 4. Conclusions

In conclusion, the identification of metabolites of eupatorin in vivo and in vitro had achieved great success firstly by means of UHPLC-Q-TOF-MS/MS combined with a powerful and efficient data acquisition and processing method. The results displayed that a total of 71 metabolites were characterized: 51 metabolites were identified in vivo (8 metabolites in the plasma, 5 metabolites in the bile, 36 metabolites in the urine and 32 metabolites in the feces), while 60 metabolites were detected in vitro (22 metabolites in the rat liver microsomes and 53 metabolites in rat intestinal flora). This study was expected to benefit future efficacy and safety studies on eupatorin and provide guidelines for intake of OS. There is no doubt that further studies are needed to confirm the impact of these metabolites on human health and safety, thus providing reasonable recommendations for the consumption of foods and drugs containing eupatorin.

## Figures and Tables

**Table 1:** Summary of metabolites of eupatorin in vivo and in vitro.

Metabolites ID	Composition	Formula	*m*/*z*	Error (ppm)	R.T. (min)	MS/MS Fragments	Clog P	Score (%)	Plasma	Bile	Urine	Feces	RLMs	RIF
M1	Loss of CH_2_ [M-H]^−^	C_17_H_14_O_7_	329.0660	−2.0	9.93	314.0427, 313.0384, 299.0188, 285.0371, 207.7129	2.26422	83.3	-	-	+^a,b^	+ ^a,b^	+^I,II^	+ ^a,b^
M2	Loss of CH_2_ [M-H]^−^	C_17_H_14_O_7_	329.0668	0.5	10.27	314.0423, 313.0344, 299.0189, 285.0393, 133.0287	2.26434	91.8	-	-	+ ^a,b^	+ ^a,b^	+ ^I,II^	+ ^a,b^
M3	Loss of CH_2_ [M-H]^−^	C_17_H_14_O_7_	329.0662	−1.4	10.79	314.0436,313.0357, 299.0204, 285.0396, 207.7166	2.51422	96.3	-	-	+ ^a,b^	+ ^a,b^	+ ^I,II^	+ ^a,b^
M4a	Loss of CH_2_ and CH_2_ [M-H]^−^	C_16_H_12_O_7_	315.0500	−3.3	7.26	300.0279, 297.1740, 269.1760, 251.1269, 133.0270	1.82034	85.4	-	-	+ ^a,b^	+ ^a,b^	+^I^	-
M4b							2.07034							
M5	Loss of CH_2_ and CH_2_ [M-H]^−^	C_16_H_12_O_7_	315.0504	−2.0	8.50	300.0275, 297.0411, 269.1755, 251.1658, 147.0821	2.18513	93.3	-	-	+ ^a,b^	+ ^a,b^	+ ^I^	-
M6a	Loss of CH_2_O [M-H]^−^	C_17_H_14_O_6_	313.0713	−1.4	13.86	298.0483, 283.0250, 221.0632, 147.0078, 117.0364	2.86048	90.9	-	-	-	+ ^a,b^	-	+ ^a,b^
M6b							3.08571							
M6c							3.33571							
M7	Loss of CH_2_O and CH_2_ [M-H]^−^	C_16_H_12_O_6_	299.0562	0.3	10.10	284.0326, 281.1787, 251.1281, 146.9687	2.74964	83.8	-	-	+ ^a^	-	+ ^I^	+ ^b^
M8	Loss of CH_2_O and CH_2_O [M-H]^−^	C_16_H_12_O_5_	283.0614	0.8	13.60	268.0379, 240.0428, 267.0306, 161.0025, 146.9655	3.30833	93.5	-	-	+ ^a,b^	+ ^b^	+ ^I^	+ ^b^
M9	Loss of O [M-H]^−^	C_18_H_16_O_6_	327.0882	2.4	4.98	308.9931, 299.1274, 281.2489, 205.0025, 146.9380	2.45814	75.3	-	-	-	-	-	+ ^b^
M10	Loss of O [M-H]^−^	C_18_H_16_O_6_	327.0872	−0.8	7.47	309.0800, 299.0957, 281.2493, 221.0452, 130.9716	3.44497	83.5	-	-	-	-	-	+ ^b^
M11	Loss of O and O [M-H]^−^	C_18_H_16_O_5_	311.0930	1.5	9.55	250.9816, 204.9868, 174.9556, 130.9658	3.19475	75.7	-	-	-	-	-	+ ^b^
M12a	Loss of O and CH_2_O [M-H]^−^	C_17_H_14_O_5_	297.0768	−0.2	7.33	267.1016, 253.0865, 175.0394, 147.0452, 145.0305	2.78433	82.1	-	-	-	-	-	+ ^b^
M12b														
M13	Loss of CO [M-H]^−^	C_17_H_16_O_6_	315.0862	−3.8	12.74	300.0633, 285.0401, 270.0144, 193.0503, 147.0445	2.84747	87.9	-	-	-	+ ^a,b^	-	+ ^a,b^
M14	Oxidation [M-H]^−^	C_18_H_16_O_8_	359.0772	−0.2	10.01	344.0542, 329.0304, 314.0064, 220.9817, 163.0384	1.79518	82.9	-	-	+ ^a,b^	+ ^a,b^	+ ^I^	+ ^a,b^
M15	Oxidation [M-H]^−^	C_18_H_16_O_8_	359.0768	−1.3	10.50	344.0529, 329.0306, 314.0061, 221.0098, 163.0368	1.84518	82.8	-	-	+ ^a,b^	+ ^a,b^	+ ^I^	+ ^a,b^
M16	Oxidation [M-H]^−^	C_18_H_16_O_8_	359.0767	−1.6	11.47	344.0536, 329.0296, 314.0066, 221.0762, 163.0019	1.86518	81.9	-	-	+ ^a,b^	+ ^a,b^	+ ^I^	+ ^a,b^
M17	Oxidation [M-H]^−^	C_18_H_16_O_8_	359.0767	−1.4	12.23	344.0542, 329.0315, 314.0085, 237.0375, 147.0130	1.87123	85.5	-	-	+ ^a,b^	+ ^a,b^	+ ^I^	+ ^a,b^
M18	Di-Oxidation [M-H]^−^	C_18_H_16_O_9_	375.0709	−3.3	9.90	329.0669, 314.0434, 299.0191, 221.1216, 178.9947	0.9644	91.2	-	-	-	+ ^a,b^	-	+ ^a^
M19	Tri-Oxidation [M-H]^−^	C_18_H_16_O_10_	391.0673	0.5	12.26	345.0869, 330.0636, 315.0393, 221.0399, 195.0289	0.25226	77.1	-	-	+ ^b^	-	-	+ ^a,b^
M20a	Demethylation and Oxidation [M-H]^−^	C_17_H_14_O_8_	345.0605	−3.0	9.43	330.0379, 301.1825, 221.1270, 149.0245, 125.0237	1.29734	84.9	-	-	+ ^a,b^	+ ^b^	+ ^I^	+ ^b^
M20b														
M21	Demethylation and Oxidation [M-H]^−^	C_17_H_14_O_8_	345.0606	−2.8	10.29	330.0384, 301.0719, 221.0028, 149.0234, 125.0311	1.59734	87.1	-	-	+ ^a,b^	+ ^b^	+ ^I^	+ ^b^
M22	Methylation [M-H]^−^	C_19_H_18_O_7_	357.0972	−2.1	10.02	342.0740, 327.0503, 312.0266, 235.0434, 147.0433	2.06632	80.6	-	-	+ ^a,b^	+ ^a,b^	-	+ ^a,b^
M23	Methylation [M-H]^−^	C_19_H_18_O_7_	357.0969	−3.1	12.86	342.0737, 327.0508, 312.0266, 221.0766, 161.0269	3.18323	78.6	-	-	+ ^a,b^	+ ^a,b^	-	+ ^a,b^
M24	Loss of O+Methylation [M-H]^−^	C_19_H_18_O_6_	341.1025	−1.5	7.15	326.1073, 311.0918, 235.0607, 130.9906, 107.0440	2.80306	82.8	-	-	-	-	-	+ ^b^
M25	Loss of O+Methylation [M-H]^−^	C_19_H_18_O_6_	341.1027	−1.2	8.79	326.0798, 311.0451, 204.9196, 161.0595, 137.0553	2.9313	82.1	-	-	-	-	-	+ ^b^
M26	Loss of CH_2_O and CH_2_O+Demethylation [M-H]^−^	C_15_H_10_O_5_	269.0459	1.3	9.89	253.0124, 241.0500, 225.0555, 133.0298, 117.0349	2.88784	95.6	+ ^a,b^	-	+ ^a,b^	-	-	+ ^a,b^
M27	Loss of CH_2_O and CH_2_+Demethylation [M-H]^−^	C_15_H_10_O_6_	285.0402	−0.9	8.45	267.0130, 241.0462, 221.0063, 177.0189, 133.0307	2.31115	90.2	-	-	-	-	-	+ ^b^
M28	Glucuronidation [M-H]^−^	C_24_H_24_O_13_	519.1140	−0.8	7.10	397.0442, 343.0822, 328.0587, 313.0346, 146.9662	−0.495	81.6	+ ^a^	+ ^b^	+ ^a,b^	-	+ ^II^	-
M29	Glucuronidation [M-H]^−^	C_24_H_24_O_13_	519.1151	1.3	8.14	343.0824, 328.0588, 323.0173, 313.0354, 221.0262	0.62193	83.1	+ ^a^	+ ^b^	+ ^a,b^	-	+ ^II^	-
M30	Demethylation and Glucuronide Conjugation [M-H]^−^	C_23_H_22_O_13_	505.0979	−1.8	7.88	329.0669, 309.0687, 299.0165, 285.0735	0.14693	81.3	-	-	+ ^a,b^	-	-	-
M31	Sulfate Conjugation [M-H]^−^	C_18_H_16_O_10_S	423.0391	0.1	8.93	343.0830, 328.0593, 313.0355, 285.0413, 147.0037	0.27032	86.1	-	+ ^a,b^	+ ^a,b^	-	-	+ ^a,b^
M32	Sulfate Conjugation [M-H]^−^	C_18_H_16_O_10_S	423.0387	−0.9	9.20	343.0836, 328.0606, 313.0371, 285.0457, 227.0084	1.38723	89.6	-	+ ^a,b^	+ ^a,b^	-	-	+ ^a,b^
M33	Loss of CH_2_+Sulfate Conjugation [M-H]^−^	C_17_H_14_O_10_S	409.0233	−0.4	9.01	329.0670, 314.0432, 299.0198, 212.0456, 132.0208	0.81223	91.5	+ ^a^	-	+ ^a,b^	-	-	-
M34	Loss of O+Sulfate Conjugation [M-H]^−^	C_18_H_16_O_9_S	407.0434	−2.1	12.65	327.0826, 301.0034, 220.9818, 131.0573	1.00706	63.3	-	-	-	-	-	+ ^b^
M35	N-Acetylation [M-H]^−^	C_20_H_18_O_8_	385.0917	−3.1	13.36	370.0729, 355.0427, 343.0846, 263.0551, 147.0513	1.49632	74.4	-	-	-	-	-	+ ^b^
M36	N-Acetylation [M-H]^−^	C_20_H_18_O_8_	385.0925	−0.9	13.90	370.0735, 355.0492, 343.0874, 221.0781, 189.0551	2.61323	77.9	-	-	-	-	-	+ ^b^
M37	Loss of O+N-Acetylation [M-H]^−^	C_20_H_18_O_7_	369.0987	2.1	13.02	327.2227, 279.0680, 263.1681, 237.1098, 130.9934	2.23306	75.3	+ ^a^	-	+ ^a,b^	+ ^a,b^	+ ^I^	+ ^a,b^
M38	Loss of O+N-Acetylation [M-H]^−^	C_20_H_18_O_7_	369.0975	−1.3	13.90	354.0755, 339.0542, 311.0594, 174.9586, 164.9289	2.3613	76.7	+ ^a^	-	+ ^a,b^	+ ^a,b^	+ ^I^	+ ^a,b^
M39	Loss of CH_2_+N-Acetylation [M-H]^−^	C_19_H_16_O_8_	371.0761	−3.2	11.45	329.0680, 314.0439, 299.0196, 220.9869, 175.0389	2.13823	76.8	-	-	-	+ ^a,b^	-	+ ^b^
M40	Loss of CH_2_O+N-Acetylation [M-H]^−^	C_19_H_16_O_7_	355.0813	−2.9	11.86	340.0587, 325.0335, 313.1107, 263.0361, 117.0329	1.64857	78.2	-	-	+ ^a,b^	+ ^a,b^	+ ^I^	+ ^a,b^
M41	Loss of CH_2_O+N-Acetylation [M-H]^−^	C_19_H_16_O_7_	355.0814	−2.6	13.15	340.0589, 325.0356, 313.0337, 221.0027, 159.1093	2.87497	78.1	-	-	+ ^a,b^	+ ^a,b^	+ ^I^	+ ^a,b^
M42	Loss of CH_2_O+Di-Acetylation of Amines [M-H]^−^	C_21_H_18_O_8_	397.0918	−2.6	15.21	382.0691, 367.0426, 313.2553, 263.0559, 159.0462	1.66306	74.8	-	-	-	+ ^b^	-	+ ^b^
M43a	Loss of CH_2_ and CH_2_+Di-Acetylation of Amines [M-H]^−^	C_20_H_16_O_9_	399.0704	−4.3	14.14	384.0497, 357.0641, 315.0523, 175.0034, 147.0316	1.56823	71.8	-	-	-	+ ^b^	-	-
M43b														
M43c														
M44a	Loss of CH_2_O and CH_2_O+Di-Acetylation of Amines [M-H]^−^	C_20_H_16_O_7_	367.0806	−4.8	15.24	283.0237, 233.1253, 202.9904, 189.0633, 159.0348	2.05475	71.9	-	-	-	+ ^a,b^	-	-
M44b							2.11133							
M44c							2.40475							
M45	Loss of O+Hydrogenation [M-H]^−^	C_18_H_18_O_6_	329.1028	−0.8	4.57	314.0861, 299.1136, 283.2624, 223.0900, 130.9874	1.71027	75.9	-	-	-	-	-	+ ^a,b^
M46	Loss of O+Hydrogenation [M-H]^−^	C_18_H_18_O_6_	329.1029	−0.5	5.14	314.0905, 299.1189, 283.1288, 207.0777, 147.0535	1.7953	76.7	-	-	-	-	-	+ ^a,b^
M47	Loss of O+Hydrogenation [M-H]^−^	C_18_H_18_O_6_	329.1029	−0.3	5.43	314.0384, 299.0986, 283.2612, 207.0618, 149.0538	2.28982	78.7	-	-	-	-	-	+ ^a,b^
M48	Loss of O+Hydrogenation [M-H]^−^	C_18_H_18_O_6_	329.1029	−0.6	9.69	314.0226, 299.0298, 283.0982, 223.0742, 133.0731	2.89116	75.8	-	-	-	-	-	+ ^a,b^
M49	Loss of O and O+Hydrogenation [M-H]^−^	C_18_H_18_O_5_	313.1080	−0.3	7.61	298.0759, 269.1191, 239.1066, 206.9936, 130.9677	2.5321	88.5	-	-	-	-	-	+ ^a,b^
M50	Loss of O and O+Hydrogenation [M-H]^−^	C_18_H_18_O_5_	313.1086	1.6	7.88	298.0767, 269.1189, 239.1061, 207.0818, 133.0654	3.02662	90.7	-	-	-	-	-	+ ^a,b^
M51	Demethylation and Hydrogenation [M-H]^−^	C_17_H_16_O_7_	331.0824	0.3	10.05	316.0595, 313.1396, 301.0343, 223.1657, 135.0450	1.70816	90.5	-	-	+ ^a,b^	-	-	+ ^a^
M52	Di-Hydrogenation [M-H]^−^	C_18_H_20_O_7_	347.1140	1.0	12.78	332.0913, 317.0676, 225.0074, 149.0591, 123.0079	0.81747	60.2	-	-	+ ^a,b^	-	-	+ ^b^
M53	Loss of O+Di-Hydrogenation [M-H]^−^	C_18_H_20_O_6_	331.1186	−0.2	3.60	301.1097, 299.1257, 285.1728, 225.0501, 133.0325	1.55427	53.1	-	-	+ ^b^	-	-	+ ^b^
M54	Loss of O+Di-Hydrogenation [M-H]^−^	C_18_H_20_O_6_	331.1185	−0.7	4.07	301.1088, 299.1304, 285.0945, 209.0813, 149.0680	1.6393	52.8	-	-	+ ^b^	-	-	+ ^b^
M55	Loss of O and O+Di-Hydrogenation [M-H]^−^	C_18_H_20_O_5_	315.1214	−1.6	6.36	285.1155, 271.1557, 269.1318, 241.1035, 133.0693	2.3761	83.6	-	-	+ ^a^	-	-	+ ^a,b^
M56	Loss of CH_2_+Di-Hydrogenation [M-H]^−^	C_17_H_18_O_7_	333.0972	−2.3	10.18	318.0813, 317.1125, 303.0579, 211.0624, 149.0642	0.32475	94.4	-	-	+ ^a,b^	-	-	-
M57	Loss of CH_2_+Di-Hydrogenation [M-H]^−^	C_17_H_18_O_7_	333.0982	0.6	10.24	315.0034, 225.2199, 179.1064, 135.1164, 109.0679	0.37127	63.2	-	-	+ ^a,b^	-	-	-
M58	Loss of CH_2_+Di-Hydrogenation [M-H]^−^	C_17_H_18_O_7_	333.0979	−0.3	10.80	315.0884, 300.0638, 285.0374, 211.0614, 149.0693	0.64475	90.6	-	-	+ ^a,b^	-	-	-
M59a	Loss of CH_2_ and CH_2_+Di-Hydrogenation [M-H]^−^	C_16_H_16_O_7_	319.0815	−2.6	8.47	301.0701, 211.0353, 197.0452, 149.0269, 135.0443	0.19855	89.0	-	-	-	+ ^a,b^	-	-
M59b							−0.1214							
M59c							0.22768							
M60	Loss of CH_2_O and CH_2_O+Di-Hydrogenation [M-H]^−^	C_16_H_16_O_5_	287.0923	−0.8	9.97	272.0689, 241.0875, 195.0653, 119.0500, 93.0325	1.35527	80.7	-	-	-	-	-	+ ^b^
M61	Loss of CH_2_O and CH_2_O+Di-Hydrogenation [M-H]^−^	C_16_H_16_O_5_	287.0927	0.5	11.07	272.0695, 241.2138, 165.0166, 149.0683, 123.0117	1.4214	85.8	-	-	-	-	-	+ ^b^
M62a	Loss of O and CH_2_O+Di-Hydrogenation [M-H]^−^	C_17_H_18_O_5_	301.1078	−1.2	7.37	271.1359, 255.0541, 241.0814, 179.0711, 149.0605	1.9972	87.9	+ ^a^	-	-	+ ^a,b^	-	-
M62b														
M63	Loss of CH_2_O and CH_2_O+Loss of Hydroxymethylene [M-H]^−^	C_15_H_10_O_4_	253.0512	2.1	7.81	225.0558, 209.0606, 161.0249, 117.0351, 101.0246	3.4753	70.9	+ ^b^	-	+ ^a,b^	-	-	+ ^b^
M64	Ketone Formation [M-H]^−^	C_18_H_14_O_8_	357.0607	−2.5	10.79	342.0374, 327.0132, 313.0304, 221.0224, 161.0174	2.17223	79.1	-	-	-	+ ^b^	+ ^I^	-
M65	Ketone Formation [M-H]^−^	C_18_H_14_O_8_	357.0609	−1.8	10.81	342.0379, 327.0146, 313.0349, 235.0241, 147.0012	2.27223	76.1	-	-	-	+ ^b^	+ ^I^	-
M66	Ketone Formation [M-H]^−^	C_18_H_14_O_8_	357.0610	−1.6	12.99	342.0385, 327.0198, 313.0421, 235.0267, 147.0503	2.52223	77.4	-	-	-	+ ^b^	+ ^I^	-
M67	Oxidation and Internal Hydrolysis [M-H]^−^	C_18_H_18_O_9_	377.0875	−0.8	12.29	362.0508, 349.0873, 239.0435, 181.0136, 139.0054	0.21452	83.4	-	-	-	+ ^a,b^	-	+ ^a,b^
M68a	Loss of CH_2_+Glycine Conjugation [M-H]^−^	C_19_H_17_NO_8_	386.0865	−4.2	10.00	329.0662, 314.0421, 299.0189, 264.1192, 147.0973	2.15391	80.8	-	-	-	+ ^b^	-	-
M68b							2.40391							
M69a	Loss of O and CH_2_O+Glycine Conjugation [M-H]^−^	C_19_H_17_NO_6_	354.0992	2.5	6.09	324.2008, 250.9077, 221.0751, 204.0387, 174.9553	2.66894	73.9	-	+ ^a^	-	-	-	-
M69b														
M70	Loss of CH_2_O+Glutamine Conjugation [M-H]^−^	C_22_H_22_N_2_O_8_	441.1302	−0.2	5.07	312.8496, 245.1021, 221.5883, 117.2304	0.99254	50.9	-	-	-	-	-	+ ^b^
M71	Loss of CH_2_ and CH_2_+Glucose Conjugation [M-H]^−^	C_22_H_22_O_12_	477.1036	−0.6	5.49	355.0661, 315.6277, 192.9548, 146.9654, 123.0795	−0.3982	52.9	-	-	-	-	-	+ ^b^

+, Detected; -, Undetected. RLMs, rat liver microsomes; RIF, rat intestinal flora. (a) Metabolites obtained by methanol precipitation protein; (b) metabolites obtained by ethyl acetate extraction; a+b metabolites obtained by methanol precipitation protein and ethyl acetate extraction. (I) Phase I metabolites obtained from liver microsomes; (II) phase II metabolites obtained from liver microsomes.
